# Methylphenidate augmentation of escitalopram to enhance adherence to antidepressant treatment: a pilot randomized controlled trial

**DOI:** 10.1186/s12888-021-03583-7

**Published:** 2021-11-19

**Authors:** Martin P. Paulus, Rayus Kuplicki, Teresa A. Victor, Hung-Wen Yeh, Sahib S. Khalsa

**Affiliations:** 1 Laureate Institute for Brain Research, 6655 S Yale Ave, Tulsa, OK 74136-3326 USA; 2 Oxley College of Health Sciences, The University of Tulsa, Tulsa, OK USA; 3 Health Services & Outcomes Research, Children’s Mercy Hospital, Kansas City, MO USA

**Keywords:** Adherence, Computational psychiatry, Stimulant, Computational neuroscience, Pharmacotherapy

## Abstract

**Background:**

Adherence to treatment, i.e. the extent to which a patient’s therapeutic engagement coincides with the prescribed treatment, is among the most important problems in mental health care. The current study investigated the influence of pairing an acute positive reinforcing dopaminergic/noradrenergic effect (methylphenidate, MPH) with a standard antidepressant on the rates of adherence to medication treatment. The primary objective of this study was to determine whether MPH + escitalopram resulted in higher rates of medication adherence relative to placebo + escitalopram.

**Methods:**

Twenty participants with moderate to severe depression were 1–1 randomized to either (1) 5 mg MPH + 10 mg escitalopram or (2) placebo + 10 mg escitalopram with the possibility for a dose increase at 4 weeks. A Bayesian analysis was conducted to evaluate the outcomes.

**Results:**

First, neither percent Pill count nor Medication Electronic Monitoring System adherence showed that MPH was superior to placebo. In fact, placebo showed slightly higher adherence rates on the primary (7.82% better than MPH) and secondary (7.07% better than MPH) outcomes. There was a less than 25% chance of MPH augmentation showing at least as good or better adherence than placebo. Second, both groups showed a significant effect of treatment on the QIDS-SR with a median effect of an 8.6-point score reduction. Third, neither subjective measures of adherence attitudes nor socio-demographic covariates had a significant influence on the primary or secondary outcome variables.

**Conclusions:**

These data do not support the use of MPH to increase adherence to antidepressant medication in individuals with moderate to severe depression.

**ClinicalTrials.gov identifier:**

NCT03388164, registered on 01/02/2018.

**Supplementary Information:**

The online version contains supplementary material available at 10.1186/s12888-021-03583-7.

## Introduction

Adherence to treatment, i.e. the extent to which the patient’s history of therapeutic engagement coincides with the prescribed treatment [1], is among the most important problems in mental health care [2, 3]. It is estimated that nearly half of all prescribed medications are not taken, that about 125,000 deaths annually are attributable to non-adherence, and that non-adherence costs are estimated between $100 and $300 billion each year [4]. Thus, non-adherence is a profound clinical challenge that incurs adverse psychosocial consequences, enormous costs, and poor outcomes that are shared by patients, family members, providers, healthcare systems, payers, and society [5]. It is estimated that only one out of five patients comply with antidepressant treatment for over 4 months [6], and that the majority of patients discontinue antidepressant medication within the first 30 days [7]. Some estimate that the median time to discontinuation of an antidepressant is about two [8] to 4 months [9]. Unfortunately, the majority of clinical trials do not report rates of adherence and for those that do only 4 out of 5 participants adhere to the treatment regimen [10], which significantly affects reported efficacy [11] and safety assessments [12]. There are slight differences between antidepressant medication classes [13], but low adherence is not limited to medication treatment for depression [14], or even mental health [15, 16]. For example, there is evidence for 25–90% adherence rates for headache treatments [17]. Moreover, non-adherence extends to other therapeutic modalities, with about 20 to 70% of individuals who initiate psychosocial mental health services discontinuing treatment prior to clinicians’ recommendations [18]. Whereas continued antidepressant treatment reduces recurrence risk [19], cardiovascular [20] and overall mortality [21, 22] as well as suicide rates [23], non-adherence has substantial consequences for the course of depression by increasing relapse [24] or recurrence [25]. Taken together, adherence to treatment must be considered as one of the most important targets of research seeking to improve therapeutic outcomes.

Building new habits is an important aspect of adherence to any treatment (medication or psychotherapy). Successfully accomplishing this motivational feat means that new treatment related behaviors need to change from a reward-related to a habit-based strategy [26]. New habits can typically be reinforced either by an immediate positive outcome (positive reinforcement), or by the avoidance of a negative outcome (negative reinforcement). With respect to depression, there are several barriers to the formation of new treatment related habits (i.e., adherence behaviors). First, depressed individuals have reduced hedonic processing mediated by the dopaminergic system [27], meaning they are less driven by what feels “good” or what is “good for the individual”. Therefore, it is often difficult to utilize tools that may typically act as positive reinforcers in other individuals (e.g. financial payment). Second, habits evolve from the strong association of a cue (situation or stimulus) with an action [28]. The strengthening of a habit relies primarily on the degree to which a behavior is expressed “automatically” (without necessary thinking about it), occurs frequently, and is clearly self-identified. Successfully cemented habits are highly efficient behaviors, and usually associated with a lack of awareness, unintentionality, and uncontrollability during their performance. Habit-formation is therefore a complex process and is likely to take weeks [29]. Prior research suggests that successful habit-formation likely involves a transition from reinforcement driven hippocampal circuit activity to stimulus-response driven basal ganglia driven circuit activity, indicating that it requires a broad shift in patterns of neural activity [30, 31]. Unfortunately, the functioning of these circuits is preferentially impaired in depressed individuals [32–35]. It is presently unclear whether interventions capable of modulating activity in these same neural circuits can improve treatment related habit formation. Third, educational interventions to enhance adherence have failed to demonstrate a clear benefit on adherence and depression outcome [36]. Fourth, a community pharmacy-based coaching program showed no intervention effect on adherence [37]. The premise of this trial was that enhancing dopaminergic activity during the initial phase of acquiring a new behavior (medication taking) would enhance the transition from action-outcome to habit-based response selection. Based on these considerations, the current study aimed to investigate the influence of pairing a pharmacological agent with an acute positive reinforcing dopaminergic/noradrenergic effect with a standard antidepressant on the rates of medication treatment adherence.

Methylphenidate (MPH) has been widely used to treat attention-deficit/hyperactivity disorder (ADHD) for the last half century [38]. The drug is a monoaminergic reuptake inhibitor, i.e. it blocks dopamine reuptake transporters [39] and increases dopamine and norepinephrine availability in the synaptic cleft [40]. MPH has strong affinity at the norepinephrine transporter [41], which exceeds its affinity for the dopamine transporter. However, its ability to increase NE is much less than that of amphetamine and it has virtually no effect on serotonin [42]. Moreover, it does not acutely produce a subjective euphoria, which has been related to its relatively weak ability to D2 receptors in the striatum [43, 44]. MPH also has weaker reinforcing properties [45] and has different pharmacokinetic profile [46] than amphetamines or cocaine, which lowers its relative abuse liability. In healthy volunteers, MPH induced improvements in working memory performance [47], which were associated with reductions in rCBF in the dorsolateral prefrontal cortex and posterior parietal cortex, and reduced the impact of emotionally arousing material on memory [48]. Interestingly, MPH has long been considered as a potential treatment for anxiety based on human and animal studies [49]. For example, MPH has been used for treating mood, behavior, and cognitive symptoms in individuals with organic brain changes [50] and traumatic brain injury [51]. In particular, several studies have shown improvement of cognitive symptoms after treatment with MPH in patients with acute brain injury [52], including speed of mental processing [53, 54], response accuracy [55], improved shifts of attention [56], caregiver ratings of attention [57], and level of depressive symptoms [58]. However, its effects on sustained attention, distractibility, and memory are less clear [59, 60].

This study aimed to determine whether the combination of a first-line antidepressant plus MPH relative to a first-line antidepressant plus placebo result in higher rates of medication adherence in individuals with moderate to severe depression. The primary objective of this study was to determine whether MPH + escitalopram results in higher rates of medication adherence relative to placebo + escitalopram. The secondary objective was to determine whether MPH + escitalopram results in greater consistency of adherence relative to placebo + escitalopram.

## Materials and methods

### Trial description

This Phase 2a randomized placebo-controlled clinical trial included two principal study arms, with an option to escalate the dose of escitalopram at week 4: (1) Study Drug A: 5 mg placebo + 10 mg escitalopram (encapsulated into one capsule) and (2) Study Drug B: 5 mg methylphenidate (MPH) + 10 mg escitalopram (encapsulated into one capsule). The pilot study was part of a two-stage design which aimed to enroll 100 subjects with depression who were to be randomized to one of the two conditions: escitalopram with placebo (*n* = 10) or escitalopram with MPH (*n* = 10) in Stage 1 and escitalopram with placebo (*n* = 40) or escitalopram with MPH (*n* = 40) in Stage 2. Participants randomized to either condition were prescribed medication over the course of 8 weeks, with in-person follow-up visits at weeks 0, 2, 4 and 8, with follow-up phone calls on weeks 1, 3 and 6. Participants randomized to each condition continued to receive usual care as defined by the treating clinician, with the restriction that participants in this group were not allowed to receive other new treatments during the study period. The trial was terminated at the end of Stage 1 due to the results of the pilot study showing the opposite direction to the hypothesis and low posterior probabilities that MPH is superior to the placebo. Therefore, Stage 2 of the study was not completed. The study was registered with the ClinicalTrials.gov Identifier NCT03388164 on 01/02/2018. The recruitment and follow-up period were between 01/02/2018 and 05/15/2018. A CONSORT diagram for the study is shown in Supplemental Fig. 1.

### Endpoints

The primary endpoint for adherence [61] was percent (%) Pill count as defined by 100 * [number of prescribed pills – number of pills remaining]/[number of days between dispensing date and return date]. The secondary endpoint for adherence was defined according to the STAR-D [62] protocol based on the Medication Electronic Monitoring System (MEMS) (Aardex), i.e. the % of doses taken on schedule within 25% of the expected time interval, defined as ±6 h from when the participant usually took the medication, estimated by fitting a line to their dosing times. Exploratory endpoints included the Beliefs About Medicines Questionnaire (BMQ [63]) – accepting/ambivalent vs. indifferent/skeptical, remission as defined by a score of 5 the QIDS-SR, and response as defined by a 50% reduction in symptoms on the QIDS-SR.

### Participants

The participants met the following inclusion criteria for study eligibility (which was closely matched on STAR-D): (1) baseline QIDS-SR ≥ 14 (moderate depression [64]), (2) age 18–65 years, (3) ability to give written informed consent, (4) MDD single-episode/recurrent, not in remission. Study demographics are described in Table 1. Individuals with the following exclusion criteria at baseline were excluded from study participation: (1) MPH-related exclusions [65] (i.e. uncontrolled hyperthyroidism, glaucoma, motor tics, monoamine oxidase inhibitor treatment, serious coronary artery disease, cardiomyopathy, serious cardiac arrhythmias, uncontrolled hypertension, peripheral vasculopathy, pregnancy), (2) diagnosis with the following mental health conditions: bipolar disorder, psychotic disorder, current substance use disorder (other than nicotine), current alcohol use disorder, (3) history of intolerability of study medications, and (4) currently taking psychiatric medications.

**Table 1:** Demographic Characteristics of the Sample

	Placebo	MPH	***p***
n	10	10	
Age (mean (sd))	31.00 (10.58)	31.50 (10.73)	0.918
Sex = Male (%)	2 (20.0)	3 (30.0)	1.000
Height (mean (sd))	65.78 (2.68)	66.50 (4.22)	0.666
Weight (mean (sd))	177.56 (50.33)	180.50 (30.93)	0.878
Race (%)			0.453
Hispanic/Latino	0 (0.0)	1 (10.0)	
Native American	1 (10.0)	2 (20.0)	
White	9 (90.0)	7 (70.0)	
Drug = MPH (%)	0 (0.0)	10 (100.0)	< 0.001
Current Smoker (%)			0.288
Every-Day Smokers	2 (22.2)	0 (0.0)	
Former Smokers	2 (22.2)	3 (30.0)	
Never Smokers	5 (55.6)	7 (70.0)	
Alcohol Use in last 30 days (mean (sd))	3.70 (9.30)	1.70 (2.79)	0.523
BMQ overuse (mean (sd))	12.50 (3.14)	11.80 (2.74)	0.602
BMQ general harm (mean (sd))	15.20 (2.10)	15.50 (2.07)	0.751
QIDS (mean (sd))	17.90 (2.73)	16.67 (2.12)	0.290

Study participants were recruited through the clinical services of the Laureate Psychiatric Clinic and Hospital (LPCH), local service providers for behavioral health and mental health, and through online, newspaper, flyer, radio or other media advertisements in the Tulsa metropolitan area. Participants were also recruited through a pre-approved Laureate Institute for Brain Research (LIBR) Screening protocol (WIRB #20101611) and through existing participants drawn from the LIBR REDCap database. Informed consent was obtained by members of the research team that received training from the principal investigator to obtain consent for this study. All participant interactions were conducted in private interview/exam rooms. Data collection occurred at a single site, the Laureate Institute for Brain Research.

Participants were free to withdraw from participation in the study at any time upon request.

The study investigator had the option to terminate participation in the study if any clinical adverse event (AE), laboratory abnormality, or other medical condition or situation occurred such that continued participation in the study would not be in the best interest of the participant, or if the participant met an exclusion criterion (either newly developed or not previously recognized) that precluded further study participation.

### Procedures

The study medications were acquired through a local compounding pharmacy in Tulsa, Oklahoma. The pharmacy dispensed and labelled the individual bottles of study medication. A study psychiatrist provided the medication to the subject at each in-house study visit. The study medications were formulated into identical compound capsules containing either (1) placebo + 10 mg escitalopram, (2) 5 mg MPH + 10 mg escitalopram, (3) placebo + 20 mg of escitalopram, or (4) 5 mg MPH + 20 mg escitalopram. The study medications were packaged in standard bottles with a MEMS tracking cap. The bottles were labeled with “Study Drug A”, “Study Drug B”, “Study Drug C” or “Study Drug D”, the number of capsules in the bottle and the administration date. At the 4-week visit, participants had the option to increase the escitalopram dose to 20 mg based on lack of clinical improvement assessed by the study psychiatrist.

The following procedures (Supplemental Fig. 2) were conducted during screening and enrollment (Week 0, which lasted 3 h): (1) obtained informed consent of potential participant verified by signature on written informed consent form for screening and study consent form; (2) recorded vital signs including temperature, heart rate, electrocardiogram, systolic and diastolic blood pressure; (3) recorded height and weight; (4) collected urine for evaluation of pregnancy status (if applicable); (5) obtained demographic information, medical history, medication history, alcohol and tobacco use history; (6) reviewed medical history and medications history to determine eligibility based on inclusion/exclusion criteria; (7) collected self-report scales; (8) obtained blood safety labs (CBC, CMP, TSH); (9) scheduled study visits for participants who were eligible and available for the duration of the study; (10) provided participants with instructions for taking the study medication; and (11) dispensed the study medication. During the follow-up period the following procedures were conducted: (1) weeks 1, 3 and 6 (phone call), the QIDS-SR and MAR questionnaires were administered via phone, (2) weeks 2 and 4 (in-person visits), the study personnel verified inclusion/exclusion criteria, recorded vital signs including temperature, heart rate, systolic and diastolic blood pressure, recorded current medications and interim medical procedures, collected self-report scales including QIDS-SR and medication adherence measures, conducted the psychiatric visit (by a board certified psychiatrist) to assess for medication side effects and treatment response, and dispensed the relevant study medication.

### Statistical rationale for pilot protocol

We proposed 20 participants for Stage 1 and 80 participants for Stage 2, both in 1:1 ratios for the two groups. In Stage 1, the proposed sample size of 10 patients per group was selected to provide an estimate of mean with margin of error equivalent to ${t}_{10-\mathrm{1,0.975}}\sqrt{1/10}=0.72$ times the standard deviation for each group at 95% confidence, or ${t}_{10+10-\mathrm{2,0.975}}\sqrt{1/10+1/10}=0.94$ times the common standard deviation for between-group difference. We assumed a 20% dropout rate for both groups at Week 8, the margin of error for the between-group difference becomes ${t}_{8+8-\mathrm{2,0.975}}\sqrt{1/8+1/8}=1.07$ times the common standard deviation. Stage 2 was designed with an interim analysis. Assuming the primary endpoint followed a Gaussian distribution, we controlled overall Type I error rate at a 0.05 two-sided level using the Hwang-Shih-DeCani spending function with *γ* parameter − 4 for the upper bound and − 2 for the binding lower bound. We proposed a sample size of 40 patients per group and anticipated a 20% dropout rate at Week 8, giving 32 patients per group or 64 in total. We set n.fix in gsDesign() to 62 instead of 64 to obtain a final sample size of 64. Because gsDesign() uses 1-sample z-statistic for continuous endpoint, the resulting effect size *θ* = 0.3558 was converted to 2-sample Hedges’ G by a factor of 2, i.e., *g* ≈ 0.71. For the maximum sample size of 64, this design led to an expected sample size of 54.7 under this alternative hypothesis, or 42.8 under the null hypothesis. Also, the variance of the primary endpoint was unknown and was proposed to be estimated from the sample, so z-tests were replaced with t-tests in analyses and the bounds were revised accordingly. Based on the quantile substitution method [66], the interim boundaries 0.40 and 2.75 for z-distribution were converted to 0.404 and 2.960 for a t-distribution with 32–2 = 30 degrees-of-freedom (df), and the critical z-value 1.96 for the final analysis was converted to 1.999 for t-distribution with 64–2 = 62 df. We evaluated these boundaries by 10,000-run simulation and confirmed that (1) under null hypothesis, there was a 65.5% chance to stop the trial for futility and a 0.3% chance to claim it successful at interim analysis, and 0.024 one-sided Type I error rate at final analysis; (2) under the alternative hypothesis with an effect size of Hedges’ G 0.714, there was only a 5.4% chance to stop the trial for futility and a 19.8% chance for success at the interim analysis, and 80.1% power at the final analysis.

To ensure balance in-group size, we used block randomization with block sizes of two. The randomization sheet was provided by the primary statistician who was not involved in the clinical assessments and given directly to the pharmacist. Investigators and participants were kept blinded during the study window until data collection and analysis were completed. All study physicians, nurses and research assistants who worked directly with participants were blinded to treatment allocation. Only the study statistician and one additional staff member who was assigned to work with the compounding pharmacy were unblinded. Participants were also asked to guess their treatment assignment and indicate their degree of certainty and reasoning at each in-person visit (Weeks 0, 2, 4 and 8).

### Bayesian statistical analysis

We evaluated the treatment effect of each outcome by a linear mixed-effects models with the following fixed effects: treatment (MPH, Placebo), visit, and treatment-by-visit interaction. The data dependency was captured by random participant intercepts. For the primary and secondary outcomes, the Visit variable included 3 levels (Week 2, 4, and 8) and the contrast of interest was the MPH-vs.-placebo difference at Week 8; for the exploratory outcome QIDS score, the Visit variable included 7 levels (Week 0, 1, 2, 3, 4, 6, and 8) and the contrasts of interest were the between-treatment differences in changes at Week 8 from Week 0, i.e. the corresponding interaction term. The. Parameters were estimated by the *rstan_lmer* function from the R rstanarm package [67] using the default and our informative priors. By default, rstanarm package applies weakly informative priors [68], where (1) the fixed-effects regression parameters follow Gaussian prior with zero mean (except the mean of the intercept is set to the sample mean) and standard deviation (SD) set to 2.5, (2) the SD of random error follows an exponential distribution with the rate parameter set to 1, (3) the variance of the random intercepts follows a gamma prior with both the shape and the scale parameters set to 1. The default setup also applies an autoscale function which automatically adjusts the SD in (1) and the exponential rate parameter in (2) based on the data scale. For the pilot study, we performed the Bayesian analysis using these default priors, and conducted sensitivity analysis for the primary and secondary outcomes with informative priors to pose our beliefs that MPS would be superior to placebo. Specifically, we (a) replaced the zero mean in (1) with values such that the adherence rates were higher in the MPH group as compared to the placebo group (96, 90, and 82% in MPH vs. 90, 79, and 66% in placebo at Week 2, 4, and 8, respectively), (b) set SD in (1) to 1, and (c) disabled the autoscale function, and left all other settings as default. For all outcomes and priors, the Hamiltonian Monte Carlo (HMC) was used for posterior sampling, and we used the default 4 chains, where each chain consisted of 2000 iterations with the first 1000 discarded as warm-up. Convergence was assessed by the Potential Scale Reduction Factor R-hat statistic. The analyses were conducted using R version 4.0.2, the rstanarm package version 2.21.1, and the tidybayes [69] package version 3.0.1.

## Results

Subjects were recruited between January and March 2018. For each group, we approached 23 participants and 23 were assessed for eligibility, 20 were randomly assigned, 10 received intended treatment in each of 2 groups, and 20 were assessed for the primary and secondary endpoint (Supplemental Fig. 1). The study was terminated in July 2018 at the end of Stage 1 based on the results of the pilot data showing the opposite direction to the hypothesis and low posterior probabilities that MPH was superior to placebo. Stage 2 was not completed. There were no serious adverse events. All results are reported based on the Intention to Treat sample, which includes all randomized participants. Participants who withdrew or were lost to follow-up were considered non-adherent for the remainder of the study schedule. Participants who withdrew from the study were retained in the analyses. Three participants withdrew due to medication side effects (2 MPH, 1 Placebo). One participant withdrew from the study due to missing study appointments, including the final appointment (MPH).

Table 1 summarizes the socio-demographic and clinical characteristics of the participants. Briefly, there were no differences across groups in any of the measures.

### Primary outcome

**Fig. 1:**
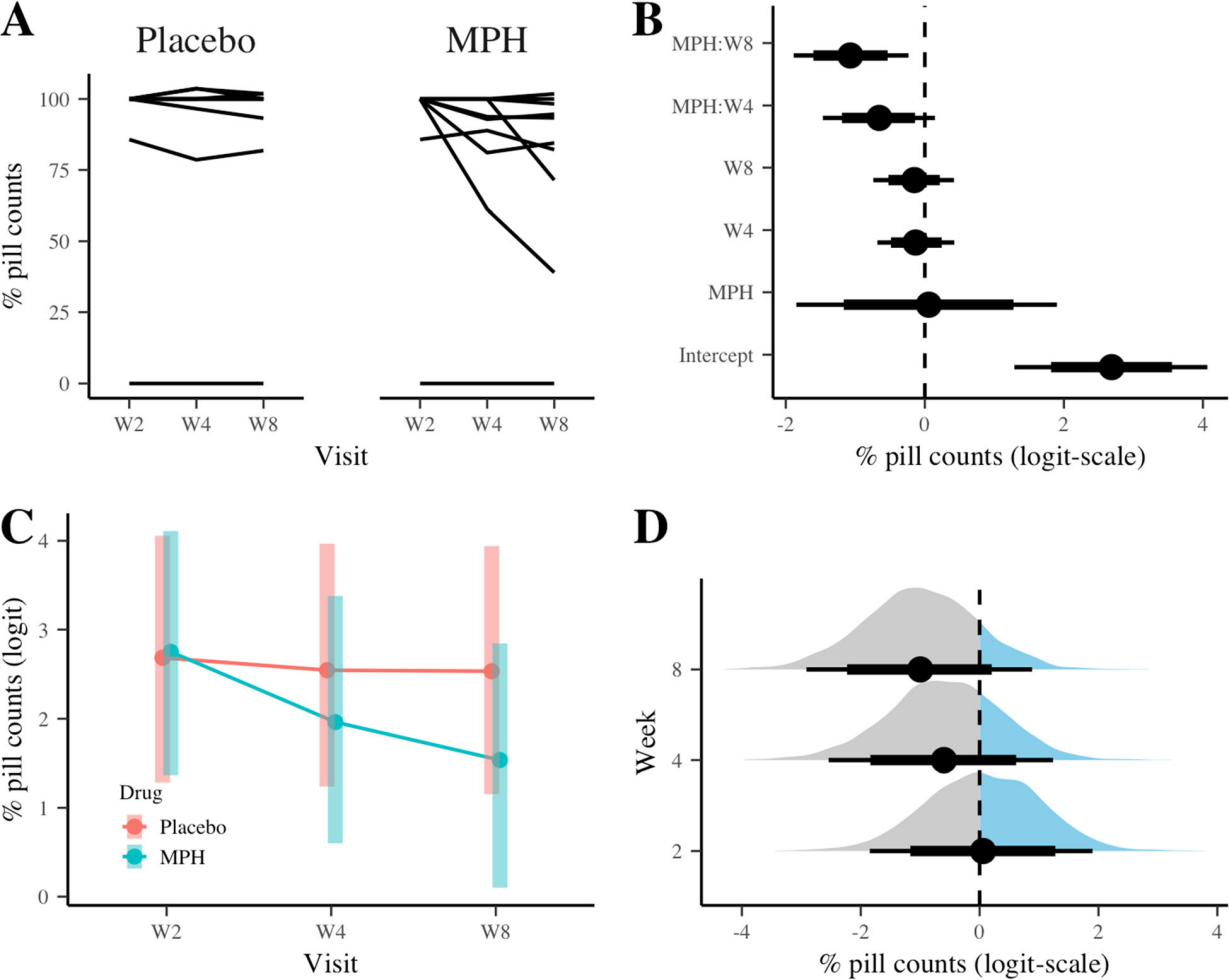
Summary of the main results for % adherence based on pill count (primary outcome). (**a**) Spaghetti plot of % pill count, (**b**) posterior distributions of the LMM fixed-effect parameters: median (dots), 80% (think bars) and 95% (thick bars) credible intervals (CI), (**c**) the estimated marginal means and 95% CI, (**d**) posterior density of between-treatment differences in adherence at Week 2, 4, and 8

Figure 1 and Table 2 summarize the main results for the primary outcome measure, i.e. % adherence based on pill count. The spaghetti plot (Fig. 1A) shows high adherence for both placebo (89%) and MPH (76%) condition, respectively, average across visits. Due to the fact that the distribution was highly skewed a logit transformation was conducted. HMC chains converged for analyses with the default weakly informative priors and our informative priors, with R-hat statistic ranging from 1.000 to 1.005 and from 1.00 to 1.010, respectively. Figure 1B shows the posterior medians (dots), 80% (thin bars) and 95% (thick bars) credible intervals for the fixed-effect parameters. The estimated marginal means (EMS, Fig. 1C) for treatment trajectories suggested lower adherence in MPH than the placebo over time, and the between-treatment contrasts (Fig. 1D) showed that there was less than a 15.0% chance (default priors) that MPH would show a higher adherence rate than placebo at Week 8 (Fig. 1D and Table 2). The same analysis using the informative priors suggested a higher but still low chance (25.0%, Supplemental R markdown file).

**Table 2:** Posterior estimates and contrasts for primary and secondary outcomes

	Default priors			Informative priors		
% pill counts (logit-scale)	Median	95% CI		Median	95% CI	
Intercept	2.69	1.29	4.06	2.52	1.39	3.66
MPH	0.06	−1.85	1.90	0.39	−0.89	1.79
Visit Week 4	−0.13	− 0.68	0.42	− 0.25	− 0.8	0.24
Visit Week 8	−0.15	−0.74	0.42	−0.3	− 0.83	0.22
MPH-by-Week 4	−0.66	−1.47	0.15	−0.5	−1.22	0.25
MPH-by-Week 8	−1.07	−1.89	−0.23	−0.92	− 1.63	−0.17
Pr (MPH > placebo)						
Week 2	0.526			0.713		
Week 4	0.265			0.434		
Week 8	0.144			0.250		
% MEMS caps (logit-scale)	Median	95% CI		Median	95% CI	
Intercept	2.75	1.41	4.2	2.51	1.3	3.54
MPH	−0.16	− 2.09	1.71	0.37	−0.98	1.72
Visit Week 4	−0.45	−1.04	0.09	−0.47	−1	0.02
Visit Week 8	−0.56	− 1.17	0.01	−0.62	− 1.13	− 0.09
MPH-by-Week 4	0.21	−0.6	1.01	0.18	−0.53	0.88
MPH-by-Week 8	−0.64	−1.45	0.19	−0.64	−1.36	0.09
Pr (MPH > placebo)						
Week 2	0.442			0.698		
Week 4	0.522			0.773		
Week 8	0.209			0.356		

### Secondary outcome

**Fig. 2:**
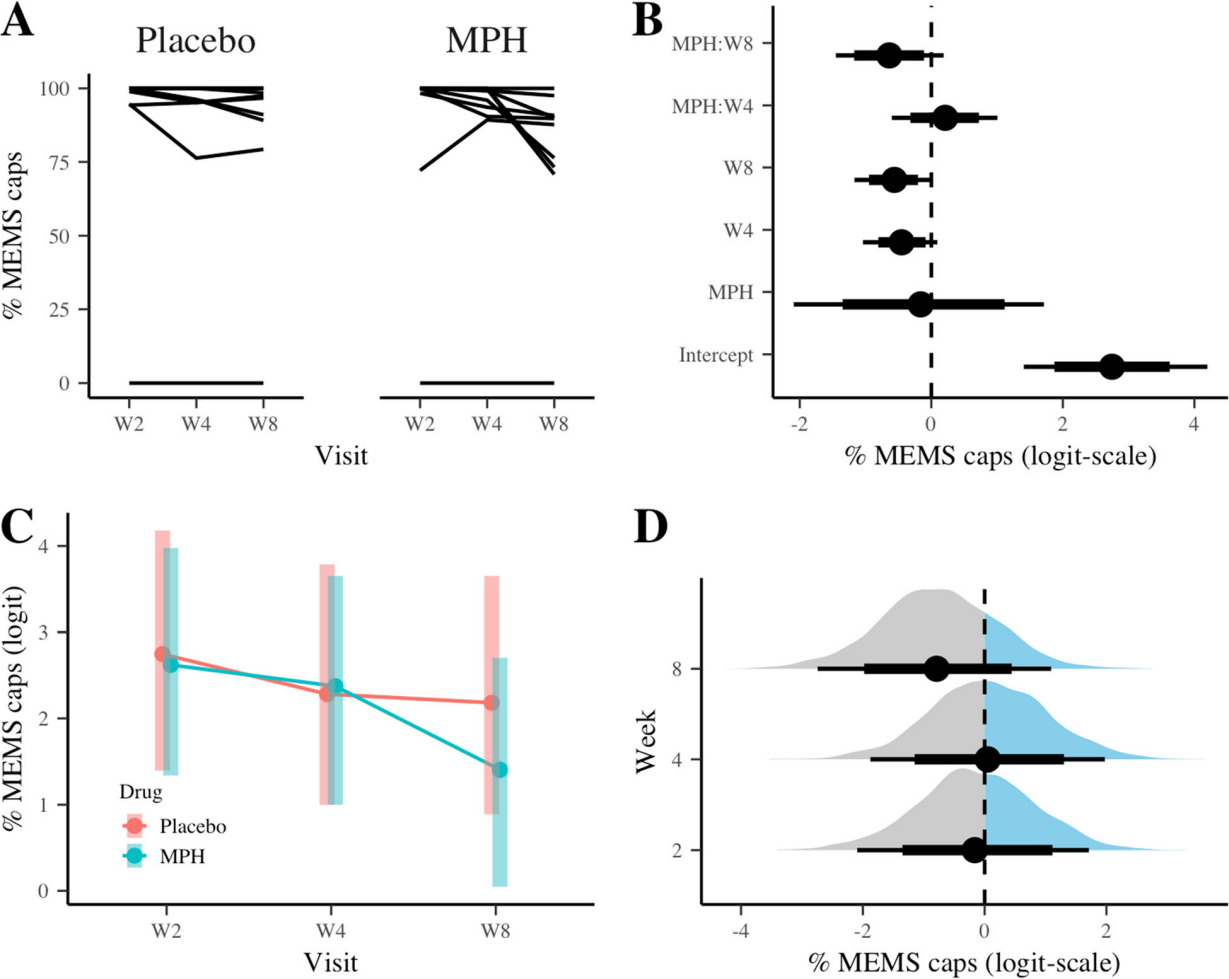
Summary of the main results for % adherence based on Medical Electronic Monitoring System (MEMS; secondary outcome). (**a**) Spaghetti plot of % MEMS caps, (**b**) posterior distributions of the LMM fixed-effect parameters: median (dots), 80% (think bars) and 95% (thick bars) credible intervals (CI), (**c**) the estimated marginal means and 95% CI, (**d**) posterior density of between-treatment differences in adherence at Week 2, 4, and 8

The secondary endpoint for adherence was based on the Medication Electronic Monitoring System (MEMS), i.e. the % of doses taken on schedule within 25% of the expected time interval. HMC chains converged as R-hat statistic ranging from 1.000 to 1.006 for both default and the informative priors. Figure 2A and B show the spaghetti plots for each treatment group and the posterior medians, 80 and 95% credible intervals (2B) for the fixed-effect parameters, respectively. The EMS were similar between treatments at Weeks 2 and 4, but slightly lower in MPH at Week 8 (Fig. 2C). Similar to the primary outcome measure, there was less than a 21.0% chance (the default priors) that MPH would show a higher adherence rate than placebo (Fig. 2D and Table 2), and the contrasts from the informative priors remained low (35.6%, Supplemental R markdown file).

### Exploratory outcome and analyses

**Fig. 3:**
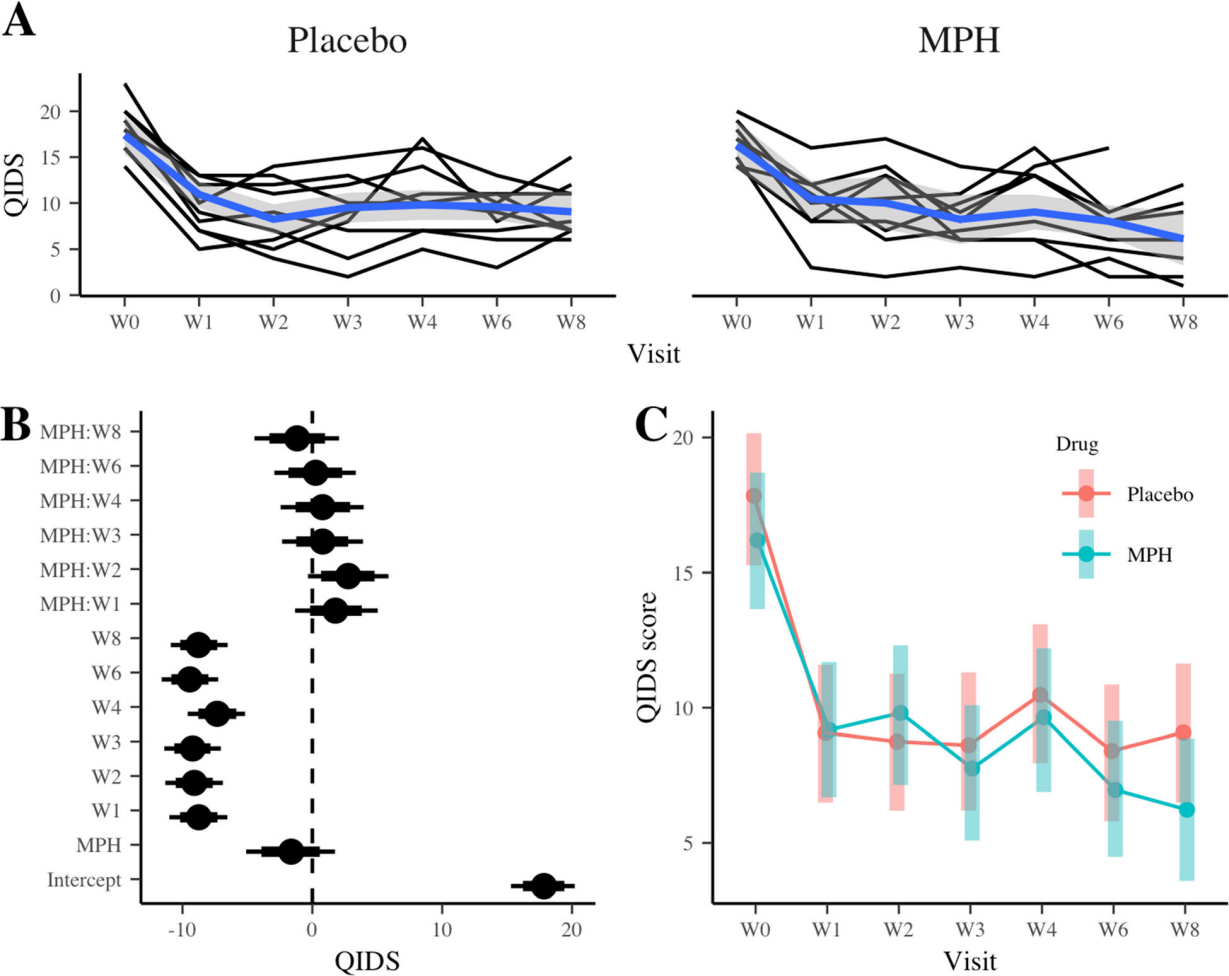
Summary of the main results for % adherence based on Quick Inventory of Depressive Symptomology (QIDS; exploratory outcome). (**a**) Spaghetti plot of QIDS score with loess smooth curves (blue lines), (**b**) posterior distributions of the LMM fixed-effect parameters: median (dots), 80% (think bars) and 95% (thick bars) credible intervals (CI), (**c**) the estimated marginal means and 95% CI

The exploratory endpoint was the remission rate according to the QIDS-SR scale for depression and the impact of the BMQ scores for individual subjects on patterns of adherence. HMC chains converged as R-hat statistic ranging from 1.000 to 1.005 for the default priors (we did not apply informative priors for this exploratory outcome). Figure 3 shows the spaghetti plot (3A), posterior medians, 80 and 95% credible intervals for the fixed-effect parameters (3B), and EMS for each treatment trajectory (3C). The spaghetti plot and the EMS suggested QIDS scores reducing over time for both treatments. At Week 8, the average QIDS changes from Week 0 was 1.2 points lower among patients receiving MPH as compared to those receiving placebo, however, the difference could be 2.1 points higher to 4.5 points lower (Table 3). Including additional covariates, i.e. age, sex, BMQ, did not change the conclusions for either the primary or the secondary outcome variable (results not shown).

**Table 3:** Posterior distributions of the fixed-effect parameters for the exploratory outcome QIDS score

Parameter	Median	95% Credible Interval	
Intercept	17.8	15.3	20.2
MPH	−1.62	−5.1	1.75
Week 1	−8.74	−11	−6.54
Week 2	−9.09	− 11.3	− 6.88
Week 3	−9.21	− 11.4	−7.04
Week 4	−7.32	−9.61	−5.18
Week 6	−9.44	−11.6	−7.25
Week 8	−8.77	−10.9	−6.52
MPH:Week 1	1.78	−1.33	5.04
MPH:Week 2	2.77	−0.34	5.87
MPH:Week 3	0.79	−2.33	3.9
MPH:Week 4	0.79	−2.45	3.95
MPH:Week 6	0.26	−2.93	3.35
MPH:Week 8	−1.18	−4.46	2.06

## Discussion

This exploratory randomized placebo-controlled clinical trial evaluated the feasibility of combining a first-line antidepressant plus MPH relative to a first-line antidepressant plus placebo in a capsule on rates of medication adherence in individuals with moderate to severe depression. The Bayesian analysis that was applied to primary, secondary and exploratory endpoints yielded two main results. First, neither % Pill count nor MEMS adherence showed that MPH was superior to placebo. In fact, placebo showed slightly higher adherence rates on the primary (7.82% better than MPH) and secondary (7.07% better than MPH) outcomes, and there was a less than 25% chance of MPH showing at least as good or better than placebo adherence. Second, both groups showed a significant effect of treatment on the QIDS-SR with a median effect of an 8.6-point reduction. Third, neither subjective measures of adherence attitudes using the BMQ nor socio-demographic covariates had a significant influence on the primary or secondary outcome variables. Taken together, these pilot data provide moderate evidence that MPH is not useful in increasing adherence to antidepressants in moderate to severely depressed individuals.

The antidepressant treatment significantly improved levels of depression in both groups and the effect size was comparable to other trials using similar interventions [70]. Thus, the lack of effect of MPH could not have been due to a lack of efficacy of the primary treatment. On the other hand, the average adherence rate for the primary outcome variable after 8 weeks of treatment was 82% (with a credible interval of 67.2–97.3%). This number is substantially higher than what has been obtained in other studies [6] and could be due to the intervention or the presence of the MEMS caps as well as the relatively short follow up period. In combination, these factors may have contributed to the difficulty in differentiating the effect of MPH versus placebo. The lack of efficacy of MPH in increasing adherence may be due to several factors. First, a higher dose of MPH may induce greater dopaminergic modulation and increase the possibility of transition from goal-directed to habitual behavior. Second, a longer follow up period may provide an opportunity for MPH to counteract increasing non-adherence. Third, selecting depressed patient based on greater anhedonia might provide a better clinical sample. Nevertheless, improving adherence in general has been a profoundly difficult challenge (e.g., see [71]).

To date, the available strategies for improving adherence include non-pharmacological approaches such as reminders, support messages, social support engagement, care team contacts, data feedback, psychoeducation, adherence-based psychotherapy, remote care delivery, secure medication storage, and contingency management [72]. Some investigators have focused on creating incentives for patients improves adherence. For example, in one study participants received a small monetary reward for keeping an appointment and taking their anti-retroviral medication. Those individuals receiving incentives were more likely to achieve 90% antiretroviral adherence compared with the control group [73]. Similarly, while financial incentives increased adherence to anti-hypertensives, none of the incentives resulted in greater use of guideline-recommended medications [74], which is consistent with minimal effects during a large post-myocardial infarction study [75]. Although there is no evidence that incentives reduce motivation to take antipsychotic medications [76], there is also minimal increase in adherence for antipsychotic medication without significant symptomatic improvement when patients are incentivized [77, 78]. While others found some increase in adherence during the incentive phase for injectable antipsychotics, this effect did not last past the incentivization period [79], which was also observed in patients with bipolar disorder [80]. In a shared incentive study, shared financial incentives for physicians and patients, but not incentives to physicians or patients alone resulted in increased statin adherence [81]. This is consistent with a review examining 22 randomized clinical trials focused on promoting adherence to antidepressants by incentivizing providers, which did not have a significant effect [82]. There is also little evidence that these interventions reduce overall mental health care costs [76]. Taken together, non-adherence to medication remains a very difficult problem that has profound effects on individual long-term outcomes.

Another finding from the current study was that participants attitudes, as measured by the BMQ, did not affect adherence rates. This questionnaire probes the ‘necessity-concerns framework’ [63], which provides a heuristic approach to understand patients’ adherence behavior. This framework proposes that the decision to take a medication is mediated by two opposing beliefs, i.e. beliefs about the necessity of taking the medication for a particular condition versus concerns about negative effects associated with taking medications. In a meta-analysis across different medical conditions and treatments, this framework significantly and differentially predicted rates of adherence [83]. For example, for each standard deviation increase in necessity beliefs, the odds of adherence increases by a factor of 1.7 and for each standard deviation increase in concerns, the odds of adherence decreases by a factor of 2 [84]. In comparison, both BMQ overuse and general harm subscores had negligible effects on both primary and secondary outcomes in this study. Moreover, depression [85] has been shown to moderate this relationship [86] with influences particularly on the concerns aspect of the framework [87]. The antidepressant-specific “necessity-minus-concerns” composite has been proposed as a predictor for adherence [88] and there is evidence that attitudes and beliefs are at least as important as side effects in predicting adherence [89]. For example, those individuals who have higher levels of concerns about antidepressant medications are less likely to be adherent [90] whereas those who report high initial expectations show greater adherence, even to placebo interventions [91]. Non-initiators have lower belief scores for impact of illness, intention to take medication, and attitude towards medication [92]. Others have found that while intentional non-adherers had higher concerns scores, unintentional non-adherers did not, which emphasizes the heterogeneity of non-adherence [93]. Moreover, those individuals who reported high necessity and high concern beliefs, i.e. were ambivalent, also showed lower levels of adherence [94]. Not surprisingly, greater non-adherence is also related to higher depression scores at follow up [95]. Interestingly, beliefs about causes of depression seem to matter little in antidepressant adherence [96]. It is likely an adherence study with more participants than were examined in this pilot study would be necessary to examine the contributions of these attitudes on adherence interventions.

The Bayesian framework provides a powerful and intuitive approach when applied to a pilot study such as this [97]. First, the Bayes factors quantitatively assessed the degree to which different models best described the observed data. Bayes factors are likelihood ratios, i.e. they describe how much more likely the observations are due to a particular model, relative to the random variation that is due to the individual differences of the participating subjects. The magnitude of the Bayes factors provides the strength of the evidence. The magnitude of the Bayes factor for the primary and secondary endpoints in this study was modest, i.e. providing “moderate” evidence [98]. Moreover, the posterior distributions give probability estimates of the outcome variable based on the best model. This enables one to not just provide a point measure, e.g. the mean or median, but also a range given by a credible interval. The results of this study showed that the credible intervals of the estimates are large and included zero, but that the probability of a desired effect by MPH was less than one in four for both primary and secondary outcomes. An important advantage is that these estimates can be used to determine whether it is useful to continue a study or whether it is preferable to stop the study. The outcomes observed in this trial, which showed a slight advantage of placebo, led us to the decision to stop the trial. This two-stage strategy could be extended to other trials and may provide a reasonable heuristic to conduct pilot studies with novel drugs to determine whether to advance to full-scale efficacy trials.

This study has several limitations. First, given the small number of participants and the single-sided nature of the study, one needs to be careful about generalizability and about the precision of the observed effects. In addition, due to large variation of estimates of measures of central tendency in small samples, one should use caution when using pilot studies to guide power calculations for study continuation [99]. Second, there is no gold standard for adherence. Although the outcome measures selected here have been proposed as reasonable adherence measures, it is well-known that different assessments can yield varied outcomes. Considering this fact, the similarity of the primary and secondary outcome effects was remarkable. Third, this pilot trial did not include a placebo treatment group. Thus, it is difficult to determine whether the effect on the exploratory outcome (QIDS-SR) was due to escitalopram or an effect of time. Therefore, we cannot speak to the efficacy of the antidepressant treatment itself.

In conclusion, the goal of this pilot study was to determine whether low-dose MPH would facilitate building the habit to take antidepressant medication and showed that it failed. Future interventions may focus on other approaches to facilitate habit formation as it relates to medication adherence.

## Supplementary Information

**Additional file 1: Supplemental Figure 1.** Consort Diagram. **Supplemental Figure 2.** Study Timeline.

## Data Availability

The full trial protocol and datasets used and/or analyzed during the current study are available from the corresponding author on reasonable request.
