# Supplementary material for: Methylphenidate augmentation of escitalopram to enhance adherence to antidepressant treatment: a pilot randomized controlled trial
Source: BMC Psychiatry. 2021 Nov 19;21:582. doi: 10.1186/s12888-021-03583-7 (PMC8603485; doi:10.1186/s12888-021-03583-7)

Supplemental Figure 1. Consort Diagram


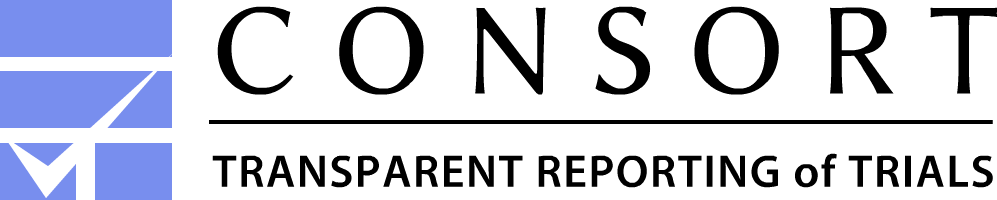


**CONSORT Flow Diagram**

## Enrollment

## Allocation

## Analysis

Analyzed (n=10)
♦ Excluded from analysis (give reasons) (n=0)

Lost to follow-up (give reasons) (n=0)

Discontinued intervention (give reasons) (n=1)
Withdrawal by subject (n=1)

Analyzed (n=10)
♦ Excluded from analysis (give reasons) (n=0)

Lost to follow-up (give reasons) (n=0)

Discontinued intervention (give reasons) (n=3)
Withdrawal by subject (n=3)

## Follow-Up

Allocated to Escitalopram + MPH (n=10)

♦ Received allocated intervention (n=10)

♦ Did not receive allocated intervention (give reasons) (n=0)

Allocated to Escitalopram + Placebo (n=10)

♦ Received allocated intervention (n=10)

♦ Did not receive allocated intervention (give reasons) (n=0)

Randomized (n=20)

Excluded (n=3)

♦  Not meeting inclusion criteria (n=2)

♦  Declined to participate (n=1)

♦  Other (n=0)

Assessed for eligibility (n=23)

Supplemental Figure 2. Study Timeline


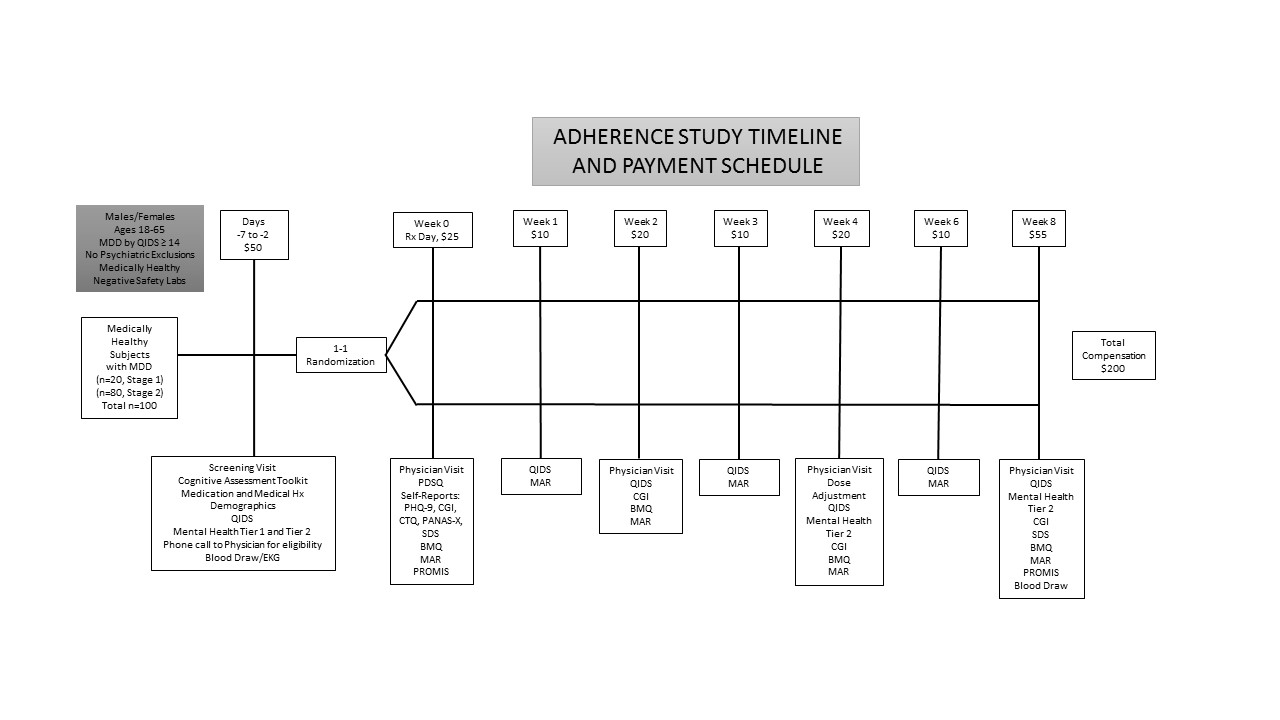

Supplement: Supplementary file 1 — Additional file 1: Supplemental Figure 1. Consort Diagram. Supplemental Figure 2. Study Timeline. [file 12888_2021_3583_MOESM1_ESM.docx]
